# Supplementary material for: Performance of five risk stratification tools for paediatric pneumonia against WHO scores using data from the PediCAP trial in sub-Saharan Africa
Source: medRxiv. 2026 Oct 30:2026.06.02.26353886. Preprint. [Version 3] doi: 10.64898/2026.06.02.26353886 (PMC13692315; doi:10.64898/2026.06.02.26353886)
Supplement: Supplementary file 1 [file media-1.docx]

# PediCAP risk scores supplementary information

## Table S1

Scores from Rees et al 2025^1^ not included in comparison

| # | Paper | Reason not suitable |
| --- | --- | --- |
| 1 | McCollum ED, et al. PLoS One. 2019^2^ | Outpatient score. Water source and sibling data not available in PediCAP. |
| 2 | King C, et al. PLoS One. 2015^3^ | Outpatient score. Number of pneumococcal conjugate vaccine (PCV) doses not available in PediCAP. |
| 3 | Xu, C, et al. Front Pediatr. 2023^4^ | Albumin, haemoglobin, blood pH data not available in PediCAP. |
| 4 | Anteneh ZA, et al. PLoS One. 2023^5^ | Full blood count data not available in PediCAP. |
| 5 | Valentania V, et al. Multidiscip Resp Med. 2021^6^ | Procalcitonin data not available in PediCAP. |
| 6 | Emukule GO, et al. PLoS One. 2014^7^ | Dehydration, night sweats and alert-voice-pain-unconscious (AVPU) scale not available in PediCAP. |
| 7 | Reed C, et al. PLoS One. 2012^8^ (The Respiratory Index of Severity in Children score for HIV-positive children) | HIV positive children mainly excluded from PediCAP due to exclusion criteria of being on long-term antibiotics for prophylaxis. |
| 8 | Mamun et al 2023. PLoS Global Public Health^9^ | Model specification was not included in the publication, and so could not be coded using PediCAP data. |

## Table S2

Components and associated risk scores included in our analysis. Component names in brackets correspond labels in supplementary figure 1.

| **Component** | **PREPARE**  (outcome: death in hospital) | **RISC-KGMU**  (outcome: death in hospital) | **PERCH**  (outcome: death in hospital + 7 days) | **RISC-Malawi**  (outcome: death in hospital) | **RISC**  (outcome: death in hospital) | **IMCI pneum-onia** | **WHO danger signs** | **N scores** |
| --- | --- | --- | --- | --- | --- | --- | --- | --- |
| **Age** (age) | 2-<6 months: 2 6 -<12 mths:1 ≥12 months: 0 |  | 1-<12 months: 2 ≥12 months: 0 |  |  |  |  | **2** |
| **Sex** (sex) | female: 1 male: 0 |  | female: 1 male: 0 | female: 1 male: 0 |  |  |  | **3** |
| **Weight-for-age z-score** (wfaz) | <-3: 3 -3 to -2: 2 ≥-2: 0 |  |  |  | <-3: 2 -3 to -2: 1 ≥-2: 0 |  |  | **3** |
| **Weight-for-height z-score** (wfhz) |  | <-3: 2 -3 to -2: 1 ≥-2: 0 | <-3: 3 -3 to -2: 2 ≥-2: 0 |  |  | <-3: 1 ≥-3: 0 | <-3: 1 ≥-3: 0 | **3** |
| **Middle upper arm circumference** (muac) |  |  |  | <11.5: 7 11.5 - < 13.5: 3 ≥ 13.5: 0 |  |  |  | **1** |
| **Duration of illness** (max dur) |  |  | <3 days: 0 ≥3 days: 2 |  |  |  |  | **1** |
| **Cyanosis** (cyanosis) | yes: 2 no: 0 |  |  |  |  |  |  | **1** |
| **Oxygen saturation** (o2sat) | <90%: 2 ≥90%: 0 | <90%: 3 ≥90% & chest indrawing: 2 ≥90%, no chest indrawing: 0 | <92%: 2 ≥92%: 0 | <90%: 7 90 - <93%: 2 ≥90%: 0 | <90%: 3 ≥90% & chest indrawing: 2 ≥90%, no chest indrawing: 0 | <90%: 1 ≥90%: 0 |  | **6** |
| **Chest indrawing** (chest indraw) |  |  | Unconscious with chest indrawing: 5 Unconscious, no chest indrawing: 2 Not unconscious: 0 |  |  | yes: 1 no: 0 |  | **5** |
| **Unconscious** (unconscious) | yes: 1 no: 0 |  |  | yes: 8 no: 0 |  | yes: 1 no: 0  (including lethargy) | yes: 1 no: 0  (including lethargy) | **5** |
| **Refusing feed**  (not feeding) |  | yes: 1 no: 0 |  |  | yes: 1 no: 0 | yes: 1 no: 0 | yes: 1 no: 0 | **4** |
| **Convulsions** (convulsions) | yes: 2 no: 0 |  |  |  |  | yes: 1 no: 0 | yes: 1 no: 0 | **3** |
| **Respiratory rate**  (resp rate) | >70 & <12 months:1 >60 & ≥ 12 months: 1 otherwise: 0 |  |  |  |  |  |  | **1** |
| **Temperature** (temp) | <35.5 °C: 3 ≥35.5 °C: 0 |  |  |  |  |  |  | **1** |
| **Cough** (cough) |  |  | yes: -1 no: 0 |  |  |  |  | **1** |
| **Grunting** (grunting) |  |  | yes: 2 no: 0 |  |  |  |  | **1** |
| **Wheeze** (wheeze) |  | yes: -2 no: 0 |  | yes: -2 no: 0 | yes: -2 no: 0 |  |  | **3** |
| **N factors** | **10** | **5** | **9** | **5** | **5** | **6** | **4** |  |
| **Minimum points** | **0** | **-2** | **-1** | **-2** | **-2** | **0** | **0** |  |
| **Maximum points** | **17** | **8** | **17** | **23** | **7** | **6** | **4** |  |
| **Range of points** | **17** | **10** | **18** | **25** | **9** | **6** | **4** |  |

## Table S3

Precision-recall estimates and 95% confidence intervals for all scores and outcomes

| score | Death in hospital | Death in hospital + 7 days | Readmission or death to d28 |
| --- | --- | --- | --- |
| PREPARE | 0.10 (0.01 - 0.20) | 0.12 (0.02 - 0.19) | 0.10 (0.05 - 0.16) |
| PREPARE: no unconsc | 0.10 (0.02 - 0.18) | 0.12 (0.02 - 0.19) | 0.10 (0.05 - 0.16) |
| RISC-KGMU | 0.20 (0.01 - 0.27) | 0.17 (0.01 - 0.25) | 0.12 (0.04 - 0.20) |
| PERCH | 0.12 (0.01 - 0.21) | 0.11 (0.01 - 0.22) | 0.10 (0.05 - 0.17) |
| PERCH: no unconsc | 0.23 (0.02 - 0.33) | 0.21 (0.02 - 0.33) | 0.14 (0.05 - 0.24) |
| RISC-Malawi | 0.11 (0.00 - 0.34) | 0.14 (0.01 - 0.26) | 0.10 (0.05 - 0.17) |
| RISC-Malawi: no unconsc | 0.12 (0.03 - 0.32) | 0.14 (0.02 - 0.27) | 0.10 (0.05 - 0.16) |
| RISC | 0.12 (0.01 - 0.18) | 0.12 (0.02 - 0.18) | 0.10 (0.05 - 0.14) |
| IMCI pneumonia | 0.14 (0.00 - 0.22) | 0.13 (0.01 - 0.22) | 0.10 (0.05 - 0.17) |
| Danger signs | 0.10 (0.00 - 0.20) | 0.09 (0.01 - 0.20) | 0.09 (0.04 - 0.15) |

## Table S4

Performance metrics for each score and threshold for the outcome of death in hospital. Cutoffs maximising Youden's index for each score are highted in gray

| **score** | **Score threshold** | **N above threshold** | **% above thres-hold** | **Number of outcomes at thres-hold** | **Number of outcomes above thres-hold** | **Sensitivity (95% CI)** | **Specificity (95% CI)** | **Positive predictive value (95% CI)** | **Negative predictive value (95% CI)** | **Positive likelihood ratio (95% CI)** | **Negative likelihood ratio (95% CI)** |
| --- | --- | --- | --- | --- | --- | --- | --- | --- | --- | --- | --- |
| PREPARE | 10 | 5 | 0.5% | 1 | 1 | 0.06 (0.00, 0.27) | 1.00 (0.99, 1.00) | 0.20 (0.01, 0.72) | 0.98 (0.97, 0.99) | 14.36 (1.69, 122.21) | 0.95 (0.85, 1.06) |
| PREPARE | 9 | 8 | 0.8% | 0 | 1 | 0.06 (0.00, 0.27) | 0.99 (0.99, 1.00) | 0.12 (0.00, 0.53) | 0.98 (0.97, 0.99) | 8.21 (1.06, 63.29) | 0.95 (0.85, 1.06) |
| PREPARE | 8 | 29 | 2.8% | 4 | 5 | 0.28 (0.10, 0.53) | 0.98 (0.97, 0.99) | 0.17 (0.06, 0.36) | 0.99 (0.98, 0.99) | 11.97 (5.15, 27.81) | 0.74 (0.56, 0.98) |
| PREPARE | 7 | 58 | 5.5% | 2 | 7 | 0.39 (0.17, 0.64) | 0.95 (0.94, 0.96) | 0.12 (0.05, 0.23) | 0.99 (0.98, 0.99) | 7.88 (4.17, 14.92) | 0.64 (0.44, 0.93) |
| PREPARE | 6 | 125 | 11.9% | 5 | 12 | 0.67 (0.41, 0.87) | 0.89 (0.87, 0.91) | 0.10 (0.05, 0.16) | 0.99 (0.99, 1.00) | 6.10 (4.21, 8.83) | 0.37 (0.19, 0.72) |
| PREPARE | 5 | 245 | 23.3% | 1 | 13 | 0.72 (0.47, 0.90) | 0.78 (0.75, 0.80) | 0.05 (0.03, 0.09) | 0.99 (0.99, 1.00) | 3.22 (2.37, 4.38) | 0.36 (0.17, 0.75) |
| PREPARE | 4 | 406 | 38.6% | 2 | 15 | 0.83 (0.59, 0.96) | 0.62 (0.59, 0.65) | 0.04 (0.02, 0.06) | 1.00 (0.99, 1.00) | 2.20 (1.77, 2.75) | 0.27 (0.10, 0.75) |
| PREPARE | 3 | 573 | 54.5% | 1 | 16 | 0.89 (0.65, 0.99) | 0.46 (0.43, 0.49) | 0.03 (0.02, 0.04) | 1.00 (0.98, 1.00) | 1.65 (1.39, 1.96) | 0.24 (0.07, 0.89) |
| PREPARE | 2 | 779 | 74.0% | 2 | 18 | 1.00 (0.81, 1.00) | 0.26 (0.24, 0.29) | 0.02 (0.01, 0.04) | 1.00 (0.99, 1.00) | 1.36 | 0 |
| PREPARE | 1 | 969 | 92.1% | 0 | 18 | 1.00 (0.81, 1.00) | 0.08 (0.06, 0.10) | 0.02 (0.01, 0.03) | 1.00 (0.96, 1.00) | 1.09 | 0 |
| PREPARE | 0 | 1052 | 100.0% | 0 | 18 | 1.00 (0.81, 1.00) | 0.00 (0.00, 0.00) | 0.02 (0.01, 0.03) |  | 1 |  |
| PREPARE: no unconsc | 10 | 1 | 0.1% | 0 | 0 | 0.00 (0.00, 0.19) | 1.00 (0.99, 1.00) | 0.00 (0.00, 0.97) | 0.98 (0.97, 0.99) | 0 | 1 |
| PREPARE: no unconsc | 9 | 6 | 0.6% | 1 | 1 | 0.06 (0.00, 0.27) | 1.00 (0.99, 1.00) | 0.17 (0.00, 0.64) | 0.98 (0.97, 0.99) | 11.49 (1.41, 93.44) | 0.95 (0.85, 1.06) |
| PREPARE: no unconsc | 8 | 17 | 1.6% | 2 | 3 | 0.17 (0.04, 0.41) | 0.99 (0.98, 0.99) | 0.18 (0.04, 0.43) | 0.99 (0.98, 0.99) | 12.31 (3.87, 39.13) | 0.84 (0.69, 1.04) |
| PREPARE: no unconsc | 7 | 36 | 3.4% | 3 | 6 | 0.33 (0.13, 0.59) | 0.97 (0.96, 0.98) | 0.17 (0.06, 0.33) | 0.99 (0.98, 0.99) | 11.49 (5.47, 24.14) | 0.69 (0.50, 0.95) |
| PREPARE: no unconsc | 6 | 87 | 8.3% | 3 | 9 | 0.50 (0.26, 0.74) | 0.92 (0.91, 0.94) | 0.10 (0.05, 0.19) | 0.99 (0.98, 1.00) | 6.63 (3.98, 11.03) | 0.54 (0.34, 0.86) |
| PREPARE: no unconsc | 5 | 175 | 16.6% | 3 | 12 | 0.67 (0.41, 0.87) | 0.84 (0.82, 0.86) | 0.07 (0.04, 0.12) | 0.99 (0.99, 1.00) | 4.23 (2.96, 6.04) | 0.40 (0.21, 0.76) |
| PREPARE: no unconsc | 4 | 331 | 31.5% | 1 | 13 | 0.72 (0.47, 0.90) | 0.69 (0.66, 0.72) | 0.04 (0.02, 0.07) | 0.99 (0.98, 1.00) | 2.35 (1.74, 3.17) | 0.40 (0.19, 0.85) |
| PREPARE: no unconsc | 3 | 482 | 45.8% | 3 | 16 | 0.89 (0.65, 0.99) | 0.55 (0.52, 0.58) | 0.03 (0.02, 0.05) | 1.00 (0.99, 1.00) | 1.97 (1.65, 2.35) | 0.20 (0.05, 0.75) |
| PREPARE: no unconsc | 2 | 701 | 66.6% | 2 | 18 | 1.00 (0.81, 1.00) | 0.34 (0.31, 0.37) | 0.03 (0.02, 0.04) | 1.00 (0.99, 1.00) | 1.51 | 0 |
| PREPARE: no unconsc | 1 | 911 | 86.6% | 0 | 18 | 1.00 (0.81, 1.00) | 0.14 (0.12, 0.16) | 0.02 (0.01, 0.03) | 1.00 (0.97, 1.00) | 1.16 | 0 |
| PREPARE: no unconsc | 0 | 1052 | 100.0% | 0 | 18 | 1.00 (0.81, 1.00) | 0.00 (0.00, 0.00) | 0.02 (0.01, 0.03) |  | 1 |  |
| RISC-KGMU | 6 | 10 | 1.0% | 5 | 5 | 0.28 (0.10, 0.53) | 1.00 (0.99, 1.00) | 0.50 (0.19, 0.81) | 0.99 (0.98, 0.99) | 57.44 (18.21, 181.18) | 0.73 (0.54, 0.97) |
| RISC-KGMU | 5 | 45 | 4.3% | 1 | 6 | 0.33 (0.13, 0.59) | 0.96 (0.95, 0.97) | 0.13 (0.05, 0.27) | 0.99 (0.98, 0.99) | 8.84 (4.29, 18.20) | 0.69 (0.50, 0.96) |
| RISC-KGMU | 4 | 232 | 22.1% | 8 | 14 | 0.78 (0.52, 0.94) | 0.79 (0.76, 0.81) | 0.06 (0.03, 0.10) | 1.00 (0.99, 1.00) | 3.69 (2.81, 4.85) | 0.28 (0.12, 0.67) |
| RISC-KGMU | 3 | 492 | 46.8% | 4 | 18 | 1.00 (0.81, 1.00) | 0.54 (0.51, 0.57) | 0.04 (0.02, 0.06) | 1.00 (0.99, 1.00) | 2.18 | 0 |
| RISC-KGMU | 2 | 796 | 75.7% | 0 | 18 | 1.00 (0.81, 1.00) | 0.25 (0.22, 0.28) | 0.02 (0.01, 0.04) | 1.00 (0.99, 1.00) | 1.33 | 0 |
| RISC-KGMU | 1 | 883 | 83.9% | 0 | 18 | 1.00 (0.81, 1.00) | 0.16 (0.14, 0.19) | 0.02 (0.01, 0.03) | 1.00 (0.98, 1.00) | 1.2 | 0 |
| RISC-KGMU | 0 | 1048 | 99.6% | 0 | 18 | 1.00 (0.81, 1.00) | 0.00 (0.00, 0.01) | 0.02 (0.01, 0.03) | 1.00 (0.40, 1.00) | 1 | 0 |
| RISC-KGMU | -1 | 1049 | 99.7% | 0 | 18 | 1.00 (0.81, 1.00) | 0.00 (0.00, 0.01) | 0.02 (0.01, 0.03) | 1.00 (0.29, 1.00) | 1 | 0 |
| RISC-KGMU | -2 | 1052 | 100.0% | 0 | 18 | 1.00 (0.81, 1.00) | 0.00 (0.00, 0.00) | 0.02 (0.01, 0.03) |  | 1 |  |
| PERCH | 16 | 2 | 0.2% | 1 | 1 | 0.06 (0.00, 0.27) | 1.00 (0.99, 1.00) | 0.50 (0.01, 0.99) | 0.98 (0.97, 0.99) | 57.44 (3.74, 882.91) | 0.95 (0.85, 1.06) |
| PERCH | 15 | 6 | 0.6% | 1 | 2 | 0.11 (0.01, 0.35) | 1.00 (0.99, 1.00) | 0.33 (0.04, 0.78) | 0.98 (0.98, 0.99) | 28.72 (5.62, 146.92) | 0.89 (0.76, 1.05) |
| PERCH | 14 | 19 | 1.8% | 1 | 3 | 0.17 (0.04, 0.41) | 0.98 (0.97, 0.99) | 0.16 (0.03, 0.40) | 0.99 (0.98, 0.99) | 10.77 (3.44, 33.74) | 0.85 (0.69, 1.04) |
| PERCH | 13 | 38 | 3.6% | 1 | 4 | 0.22 (0.06, 0.48) | 0.97 (0.95, 0.98) | 0.11 (0.03, 0.25) | 0.99 (0.98, 0.99) | 6.76 (2.68, 17.05) | 0.80 (0.63, 1.03) |
| PERCH | 12 | 73 | 6.9% | 3 | 7 | 0.39 (0.17, 0.64) | 0.94 (0.92, 0.95) | 0.10 (0.04, 0.19) | 0.99 (0.98, 0.99) | 6.09 (3.26, 11.38) | 0.65 (0.45, 0.94) |
| PERCH | 11 | 104 | 9.9% | 2 | 9 | 0.50 (0.26, 0.74) | 0.91 (0.89, 0.93) | 0.09 (0.04, 0.16) | 0.99 (0.98, 1.00) | 5.44 (3.30, 8.97) | 0.55 (0.35, 0.87) |
| PERCH | 10 | 169 | 16.1% | 1 | 10 | 0.56 (0.31, 0.78) | 0.85 (0.82, 0.87) | 0.06 (0.03, 0.11) | 0.99 (0.98, 1.00) | 3.61 (2.33, 5.59) | 0.53 (0.31, 0.88) |
| PERCH | 9 | 228 | 21.7% | 0 | 10 | 0.56 (0.31, 0.78) | 0.79 (0.76, 0.81) | 0.04 (0.02, 0.08) | 0.99 (0.98, 1.00) | 2.64 (1.71, 4.05) | 0.56 (0.34, 0.94) |
| PERCH | 8 | 323 | 30.7% | 4 | 14 | 0.78 (0.52, 0.94) | 0.70 (0.67, 0.73) | 0.04 (0.02, 0.07) | 0.99 (0.99, 1.00) | 2.60 (2.00, 3.39) | 0.32 (0.13, 0.75) |
| PERCH | 7 | 412 | 39.2% | 2 | 16 | 0.89 (0.65, 0.99) | 0.62 (0.59, 0.65) | 0.04 (0.02, 0.06) | 1.00 (0.99, 1.00) | 2.32 (1.94, 2.78) | 0.18 (0.05, 0.67) |
| PERCH | 6 | 540 | 51.3% | 0 | 16 | 0.89 (0.65, 0.99) | 0.49 (0.46, 0.52) | 0.03 (0.02, 0.05) | 1.00 (0.99, 1.00) | 1.75 (1.47, 2.09) | 0.23 (0.06, 0.83) |
| PERCH | 5 | 629 | 59.8% | 1 | 17 | 0.94 (0.73, 1.00) | 0.41 (0.38, 0.44) | 0.03 (0.02, 0.04) | 1.00 (0.99, 1.00) | 1.60 (1.41, 1.80) | 0.14 (0.02, 0.92) |
| PERCH | 4 | 734 | 69.8% | 0 | 17 | 0.94 (0.73, 1.00) | 0.31 (0.28, 0.34) | 0.02 (0.01, 0.04) | 1.00 (0.98, 1.00) | 1.36 (1.21, 1.53) | 0.18 (0.03, 1.22) |
| PERCH | 3 | 808 | 76.8% | 1 | 18 | 1.00 (0.81, 1.00) | 0.24 (0.21, 0.26) | 0.02 (0.01, 0.03) | 1.00 (0.98, 1.00) | 1.31 | 0 |
| PERCH | 2 | 878 | 83.5% | 0 | 18 | 1.00 (0.81, 1.00) | 0.17 (0.15, 0.19) | 0.02 (0.01, 0.03) | 1.00 (0.98, 1.00) | 1.2 | 0 |
| PERCH | 1 | 966 | 91.8% | 0 | 18 | 1.00 (0.81, 1.00) | 0.08 (0.07, 0.10) | 0.02 (0.01, 0.03) | 1.00 (0.96, 1.00) | 1.09 | 0 |
| PERCH | 0 | 1008 | 95.8% | 0 | 18 | 1.00 (0.81, 1.00) | 0.04 (0.03, 0.06) | 0.02 (0.01, 0.03) | 1.00 (0.92, 1.00) | 1.04 | 0 |
| PERCH | -1 | 1052 | 100.0% | 0 | 18 | 1.00 (0.81, 1.00) | 0.00 (0.00, 0.00) | 0.02 (0.01, 0.03) |  | 1 |  |
| PERCH: no unconsc | 11 | 4 | 0.4% | 3 | 3 | 0.17 (0.04, 0.41) | 1.00 (0.99, 1.00) | 0.75 (0.19, 0.99) | 0.99 (0.98, 0.99) | 172.33 (18.82, 1578.38) | 0.83 (0.68, 1.03) |
| PERCH: no unconsc | 10 | 8 | 0.8% | 1 | 4 | 0.22 (0.06, 0.48) | 1.00 (0.99, 1.00) | 0.50 (0.16, 0.84) | 0.99 (0.98, 0.99) | 57.44 (15.57, 211.89) | 0.78 (0.61, 1.00) |
| PERCH: no unconsc | 9 | 28 | 2.7% | 1 | 5 | 0.28 (0.10, 0.53) | 0.98 (0.97, 0.99) | 0.18 (0.06, 0.37) | 0.99 (0.98, 0.99) | 12.49 (5.35, 29.14) | 0.74 (0.55, 0.98) |
| PERCH: no unconsc | 8 | 68 | 6.5% | 3 | 8 | 0.44 (0.22, 0.69) | 0.94 (0.93, 0.96) | 0.12 (0.05, 0.22) | 0.99 (0.98, 1.00) | 7.66 (4.32, 13.57) | 0.59 (0.39, 0.89) |
| PERCH: no unconsc | 7 | 144 | 13.7% | 5 | 13 | 0.72 (0.47, 0.90) | 0.87 (0.85, 0.89) | 0.09 (0.05, 0.15) | 0.99 (0.99, 1.00) | 5.70 (4.11, 7.91) | 0.32 (0.15, 0.67) |
| PERCH: no unconsc | 6 | 238 | 22.6% | 0 | 13 | 0.72 (0.47, 0.90) | 0.78 (0.76, 0.81) | 0.05 (0.03, 0.09) | 0.99 (0.99, 1.00) | 3.32 (2.44, 4.52) | 0.36 (0.17, 0.75) |
| PERCH: no unconsc | 5 | 373 | 35.5% | 2 | 15 | 0.83 (0.59, 0.96) | 0.65 (0.62, 0.68) | 0.04 (0.02, 0.07) | 1.00 (0.99, 1.00) | 2.41 (1.93, 3.01) | 0.25 (0.09, 0.72) |
| PERCH: no unconsc | 4 | 512 | 48.7% | 0 | 15 | 0.83 (0.59, 0.96) | 0.52 (0.49, 0.55) | 0.03 (0.02, 0.05) | 0.99 (0.98, 1.00) | 1.73 (1.40, 2.15) | 0.32 (0.11, 0.90) |
| PERCH: no unconsc | 3 | 658 | 62.5% | 3 | 18 | 1.00 (0.81, 1.00) | 0.38 (0.35, 0.41) | 0.03 (0.02, 0.04) | 1.00 (0.99, 1.00) | 1.62 | 0 |
| PERCH: no unconsc | 2 | 781 | 74.2% | 0 | 18 | 1.00 (0.81, 1.00) | 0.26 (0.24, 0.29) | 0.02 (0.01, 0.04) | 1.00 (0.99, 1.00) | 1.36 | 0 |
| PERCH: no unconsc | 1 | 933 | 88.7% | 0 | 18 | 1.00 (0.81, 1.00) | 0.12 (0.10, 0.14) | 0.02 (0.01, 0.03) | 1.00 (0.97, 1.00) | 1.13 | 0 |
| PERCH: no unconsc | 0 | 991 | 94.2% | 0 | 18 | 1.00 (0.81, 1.00) | 0.06 (0.05, 0.08) | 0.02 (0.01, 0.03) | 1.00 (0.94, 1.00) | 1.06 | 0 |
| PERCH: no unconsc | -1 | 1052 | 100.0% | 0 | 18 | 1.00 (0.81, 1.00) | 0.00 (0.00, 0.00) | 0.02 (0.01, 0.03) |  | 1 |  |
| RISC-Malawi | 23 | 5 | 0.5% | 1 | 1 | 0.06 (0.00, 0.27) | 1.00 (0.99, 1.00) | 0.20 (0.01, 0.72) | 0.98 (0.97, 0.99) | 14.36 (1.69, 122.21) | 0.95 (0.85, 1.06) |
| RISC-Malawi | 22 | 11 | 1.0% | 2 | 3 | 0.17 (0.04, 0.41) | 0.99 (0.98, 1.00) | 0.27 (0.06, 0.61) | 0.99 (0.98, 0.99) | 21.54 (6.22, 74.62) | 0.84 (0.68, 1.03) |
| RISC-Malawi | 21 | 14 | 1.3% | 0 | 3 | 0.17 (0.04, 0.41) | 0.99 (0.98, 0.99) | 0.21 (0.05, 0.51) | 0.99 (0.98, 0.99) | 15.67 (4.77, 51.42) | 0.84 (0.69, 1.04) |
| RISC-Malawi | 20 | 15 | 1.4% | 0 | 3 | 0.17 (0.04, 0.41) | 0.99 (0.98, 0.99) | 0.20 (0.04, 0.48) | 0.99 (0.98, 0.99) | 14.36 (4.43, 46.56) | 0.84 (0.69, 1.04) |
| RISC-Malawi | 19 | 29 | 2.8% | 1 | 4 | 0.22 (0.06, 0.48) | 0.98 (0.96, 0.98) | 0.14 (0.04, 0.32) | 0.99 (0.98, 0.99) | 9.19 (3.57, 23.70) | 0.80 (0.62, 1.02) |
| RISC-Malawi | 18 | 61 | 5.8% | 4 | 8 | 0.44 (0.22, 0.69) | 0.95 (0.93, 0.96) | 0.13 (0.06, 0.24) | 0.99 (0.98, 1.00) | 8.67 (4.86, 15.48) | 0.59 (0.39, 0.89) |
| RISC-Malawi | 17 | 63 | 6.0% | 0 | 8 | 0.44 (0.22, 0.69) | 0.95 (0.93, 0.96) | 0.13 (0.06, 0.23) | 0.99 (0.98, 1.00) | 8.36 (4.69, 14.88) | 0.59 (0.39, 0.89) |
| RISC-Malawi | 16 | 100 | 9.5% | 0 | 8 | 0.44 (0.22, 0.69) | 0.91 (0.89, 0.93) | 0.08 (0.04, 0.15) | 0.99 (0.98, 0.99) | 5.00 (2.88, 8.68) | 0.61 (0.40, 0.92) |
| RISC-Malawi | 15 | 157 | 14.9% | 3 | 11 | 0.61 (0.36, 0.83) | 0.86 (0.84, 0.88) | 0.07 (0.04, 0.12) | 0.99 (0.98, 1.00) | 4.33 (2.91, 6.44) | 0.45 (0.25, 0.81) |
| RISC-Malawi | 14 | 166 | 15.8% | 0 | 11 | 0.61 (0.36, 0.83) | 0.85 (0.83, 0.87) | 0.07 (0.03, 0.12) | 0.99 (0.98, 1.00) | 4.08 (2.74, 6.06) | 0.46 (0.26, 0.82) |
| RISC-Malawi | 13 | 180 | 17.1% | 0 | 11 | 0.61 (0.36, 0.83) | 0.84 (0.81, 0.86) | 0.06 (0.03, 0.11) | 0.99 (0.98, 1.00) | 3.74 (2.52, 5.54) | 0.46 (0.26, 0.83) |
| RISC-Malawi | 12 | 202 | 19.2% | 0 | 11 | 0.61 (0.36, 0.83) | 0.82 (0.79, 0.84) | 0.05 (0.03, 0.10) | 0.99 (0.98, 1.00) | 3.31 (2.24, 4.89) | 0.48 (0.27, 0.85) |
| RISC-Malawi | 11 | 264 | 25.1% | 3 | 14 | 0.78 (0.52, 0.94) | 0.76 (0.73, 0.78) | 0.05 (0.03, 0.09) | 0.99 (0.99, 1.00) | 3.22 (2.46, 4.21) | 0.29 (0.12, 0.70) |
| RISC-Malawi | 10 | 310 | 29.5% | 1 | 15 | 0.83 (0.59, 0.96) | 0.71 (0.69, 0.74) | 0.05 (0.03, 0.08) | 1.00 (0.99, 1.00) | 2.92 (2.33, 3.67) | 0.23 (0.08, 0.66) |
| RISC-Malawi | 9 | 396 | 37.6% | 0 | 15 | 0.83 (0.59, 0.96) | 0.63 (0.60, 0.66) | 0.04 (0.02, 0.06) | 1.00 (0.99, 1.00) | 2.26 (1.81, 2.82) | 0.26 (0.09, 0.74) |
| RISC-Malawi | 8 | 544 | 51.7% | 0 | 15 | 0.83 (0.59, 0.96) | 0.49 (0.46, 0.52) | 0.03 (0.02, 0.05) | 0.99 (0.98, 1.00) | 1.63 (1.31, 2.02) | 0.34 (0.12, 0.96) |
| RISC-Malawi | 7 | 612 | 58.2% | 1 | 16 | 0.89 (0.65, 0.99) | 0.42 (0.39, 0.45) | 0.03 (0.02, 0.04) | 1.00 (0.98, 1.00) | 1.54 (1.30, 1.83) | 0.26 (0.07, 0.97) |
| RISC-Malawi | 6 | 643 | 61.1% | 0 | 16 | 0.89 (0.65, 0.99) | 0.39 (0.36, 0.42) | 0.02 (0.01, 0.04) | 1.00 (0.98, 1.00) | 1.47 (1.24, 1.74) | 0.28 (0.08, 1.04) |
| RISC-Malawi | 5 | 660 | 62.7% | 0 | 16 | 0.89 (0.65, 0.99) | 0.38 (0.35, 0.41) | 0.02 (0.01, 0.04) | 0.99 (0.98, 1.00) | 1.43 (1.20, 1.69) | 0.29 (0.08, 1.09) |
| RISC-Malawi | 4 | 701 | 66.6% | 0 | 16 | 0.89 (0.65, 0.99) | 0.34 (0.31, 0.37) | 0.02 (0.01, 0.04) | 0.99 (0.98, 1.00) | 1.34 (1.13, 1.59) | 0.33 (0.09, 1.22) |
| RISC-Malawi | 3 | 741 | 70.4% | 1 | 17 | 0.94 (0.73, 1.00) | 0.30 (0.27, 0.33) | 0.02 (0.01, 0.04) | 1.00 (0.98, 1.00) | 1.35 (1.20, 1.52) | 0.19 (0.03, 1.25) |
| RISC-Malawi | 2 | 761 | 72.3% | 0 | 17 | 0.94 (0.73, 1.00) | 0.28 (0.25, 0.31) | 0.02 (0.01, 0.04) | 1.00 (0.98, 1.00) | 1.31 (1.17, 1.48) | 0.20 (0.03, 1.33) |
| RISC-Malawi | 1 | 851 | 80.9% | 0 | 17 | 0.94 (0.73, 1.00) | 0.19 (0.17, 0.22) | 0.02 (0.01, 0.03) | 1.00 (0.97, 1.00) | 1.17 (1.04, 1.31) | 0.29 (0.04, 1.94) |
| RISC-Malawi | 0 | 939 | 89.3% | 1 | 18 | 1.00 (0.81, 1.00) | 0.11 (0.09, 0.13) | 0.02 (0.01, 0.03) | 1.00 (0.97, 1.00) | 1.12 | 0 |
| RISC-Malawi | -1 | 987 | 93.8% | 0 | 18 | 1.00 (0.81, 1.00) | 0.06 (0.05, 0.08) | 0.02 (0.01, 0.03) | 1.00 (0.94, 1.00) | 1.07 | 0 |
| RISC-Malawi | -2 | 1052 | 100.0% | 0 | 18 | 1.00 (0.81, 1.00) | 0.00 (0.00, 0.00) | 0.02 (0.01, 0.03) |  | 1 |  |
| RISC-Malawi: no unconsc | 15 | 15 | 1.4% | 3 | 3 | 0.17 (0.04, 0.41) | 0.99 (0.98, 0.99) | 0.20 (0.04, 0.48) | 0.99 (0.98, 0.99) | 14.36 (4.43, 46.56) | 0.84 (0.69, 1.04) |
| RISC-Malawi: no unconsc | 14 | 25 | 2.4% | 2 | 5 | 0.28 (0.10, 0.53) | 0.98 (0.97, 0.99) | 0.20 (0.07, 0.41) | 0.99 (0.98, 0.99) | 14.36 (6.06, 34.01) | 0.74 (0.55, 0.98) |
| RISC-Malawi: no unconsc | 13 | 29 | 2.8% | 0 | 5 | 0.28 (0.10, 0.53) | 0.98 (0.97, 0.99) | 0.17 (0.06, 0.36) | 0.99 (0.98, 0.99) | 11.97 (5.15, 27.81) | 0.74 (0.56, 0.98) |
| RISC-Malawi: no unconsc | 12 | 30 | 2.9% | 0 | 5 | 0.28 (0.10, 0.53) | 0.98 (0.96, 0.98) | 0.17 (0.06, 0.35) | 0.99 (0.98, 0.99) | 11.49 (4.96, 26.60) | 0.74 (0.56, 0.99) |
| RISC-Malawi: no unconsc | 11 | 69 | 6.6% | 3 | 8 | 0.44 (0.22, 0.69) | 0.94 (0.92, 0.95) | 0.12 (0.05, 0.22) | 0.99 (0.98, 1.00) | 7.53 (4.26, 13.33) | 0.59 (0.39, 0.89) |
| RISC-Malawi: no unconsc | 10 | 128 | 12.2% | 5 | 13 | 0.72 (0.47, 0.90) | 0.89 (0.87, 0.91) | 0.10 (0.06, 0.17) | 0.99 (0.99, 1.00) | 6.49 (4.65, 9.07) | 0.31 (0.15, 0.66) |
| RISC-Malawi: no unconsc | 9 | 137 | 13.0% | 0 | 13 | 0.72 (0.47, 0.90) | 0.88 (0.86, 0.90) | 0.09 (0.05, 0.16) | 0.99 (0.99, 1.00) | 6.02 (4.33, 8.38) | 0.32 (0.15, 0.67) |
| RISC-Malawi: no unconsc | 8 | 218 | 20.7% | 0 | 13 | 0.72 (0.47, 0.90) | 0.80 (0.78, 0.83) | 0.06 (0.03, 0.10) | 0.99 (0.99, 1.00) | 3.64 (2.67, 4.97) | 0.35 (0.16, 0.73) |
| RISC-Malawi: no unconsc | 7 | 322 | 30.6% | 2 | 15 | 0.83 (0.59, 0.96) | 0.70 (0.67, 0.73) | 0.05 (0.03, 0.08) | 1.00 (0.99, 1.00) | 2.81 (2.24, 3.52) | 0.24 (0.08, 0.67) |
| RISC-Malawi: no unconsc | 6 | 345 | 32.8% | 0 | 15 | 0.83 (0.59, 0.96) | 0.68 (0.65, 0.71) | 0.04 (0.02, 0.07) | 1.00 (0.99, 1.00) | 2.61 (2.09, 3.27) | 0.24 (0.09, 0.69) |
| RISC-Malawi: no unconsc | 5 | 375 | 35.6% | 0 | 15 | 0.83 (0.59, 0.96) | 0.65 (0.62, 0.68) | 0.04 (0.02, 0.07) | 1.00 (0.99, 1.00) | 2.39 (1.92, 2.99) | 0.26 (0.09, 0.72) |
| RISC-Malawi: no unconsc | 4 | 438 | 41.6% | 0 | 15 | 0.83 (0.59, 0.96) | 0.59 (0.56, 0.62) | 0.03 (0.02, 0.06) | 1.00 (0.99, 1.00) | 2.04 (1.64, 2.54) | 0.28 (0.10, 0.79) |
| RISC-Malawi: no unconsc | 3 | 515 | 49.0% | 2 | 17 | 0.94 (0.73, 1.00) | 0.52 (0.49, 0.55) | 0.03 (0.02, 0.05) | 1.00 (0.99, 1.00) | 1.96 (1.72, 2.23) | 0.11 (0.02, 0.72) |
| RISC-Malawi: no unconsc | 2 | 554 | 52.7% | 0 | 17 | 0.94 (0.73, 1.00) | 0.48 (0.45, 0.51) | 0.03 (0.02, 0.05) | 1.00 (0.99, 1.00) | 1.82 (1.60, 2.06) | 0.12 (0.02, 0.78) |
| RISC-Malawi: no unconsc | 1 | 723 | 68.7% | 0 | 17 | 0.94 (0.73, 1.00) | 0.32 (0.29, 0.35) | 0.02 (0.01, 0.04) | 1.00 (0.98, 1.00) | 1.38 (1.23, 1.56) | 0.18 (0.03, 1.18) |
| RISC-Malawi: no unconsc | 0 | 915 | 87.0% | 1 | 18 | 1.00 (0.81, 1.00) | 0.13 (0.11, 0.15) | 0.02 (0.01, 0.03) | 1.00 (0.97, 1.00) | 1.15 | 0 |
| RISC-Malawi: no unconsc | -1 | 974 | 92.6% | 0 | 18 | 1.00 (0.81, 1.00) | 0.08 (0.06, 0.09) | 0.02 (0.01, 0.03) | 1.00 (0.95, 1.00) | 1.08 | 0 |
| RISC-Malawi: no unconsc | -2 | 1052 | 100.0% | 0 | 18 | 1.00 (0.81, 1.00) | 0.00 (0.00, 0.00) | 0.02 (0.01, 0.03) |  | 1 |  |
| RISC | 6 | 23 | 2.2% | 5 | 5 | 0.28 (0.10, 0.53) | 0.98 (0.97, 0.99) | 0.22 (0.07, 0.44) | 0.99 (0.98, 0.99) | 15.96 (6.66, 38.26) | 0.74 (0.55, 0.98) |
| RISC | 5 | 72 | 6.8% | 3 | 8 | 0.44 (0.22, 0.69) | 0.94 (0.92, 0.95) | 0.11 (0.05, 0.21) | 0.99 (0.98, 1.00) | 7.18 (4.07, 12.68) | 0.59 (0.39, 0.90) |
| RISC | 4 | 264 | 25.1% | 6 | 14 | 0.78 (0.52, 0.94) | 0.76 (0.73, 0.78) | 0.05 (0.03, 0.09) | 0.99 (0.99, 1.00) | 3.22 (2.46, 4.21) | 0.29 (0.12, 0.70) |
| RISC | 3 | 524 | 49.8% | 4 | 18 | 1.00 (0.81, 1.00) | 0.51 (0.48, 0.54) | 0.03 (0.02, 0.05) | 1.00 (0.99, 1.00) | 2.04 | 0 |
| RISC | 2 | 794 | 75.5% | 0 | 18 | 1.00 (0.81, 1.00) | 0.25 (0.22, 0.28) | 0.02 (0.01, 0.04) | 1.00 (0.99, 1.00) | 1.33 | 0 |
| RISC | 1 | 881 | 83.7% | 0 | 18 | 1.00 (0.81, 1.00) | 0.17 (0.14, 0.19) | 0.02 (0.01, 0.03) | 1.00 (0.98, 1.00) | 1.2 | 0 |
| RISC | 0 | 1048 | 99.6% | 0 | 18 | 1.00 (0.81, 1.00) | 0.00 (0.00, 0.01) | 0.02 (0.01, 0.03) | 1.00 (0.40, 1.00) | 1 | 0 |
| RISC | -1 | 1050 | 99.8% | 0 | 18 | 1.00 (0.81, 1.00) | 0.00 (0.00, 0.01) | 0.02 (0.01, 0.03) | 1.00 (0.16, 1.00) | 1 | 0 |
| RISC | -2 | 1052 | 100.0% | 0 | 18 | 1.00 (0.81, 1.00) | 0.00 (0.00, 0.00) | 0.02 (0.01, 0.03) |  | 1 |  |
| IMCI pneumonia | 5 | 21 | 2.0% | 6 | 6 | 0.33 (0.13, 0.59) | 0.99 (0.98, 0.99) | 0.29 (0.11, 0.52) | 0.99 (0.98, 0.99) | 22.98 (10.08, 52.39) | 0.68 (0.49, 0.94) |
| IMCI pneumonia | 4 | 117 | 11.1% | 4 | 10 | 0.56 (0.31, 0.78) | 0.90 (0.88, 0.91) | 0.09 (0.04, 0.15) | 0.99 (0.98, 1.00) | 5.37 (3.42, 8.42) | 0.50 (0.30, 0.83) |
| IMCI pneumonia | 3 | 360 | 34.2% | 4 | 14 | 0.78 (0.52, 0.94) | 0.67 (0.64, 0.69) | 0.04 (0.02, 0.06) | 0.99 (0.99, 1.00) | 2.32 (1.79, 3.02) | 0.33 (0.14, 0.79) |
| IMCI pneumonia | 2 | 742 | 70.5% | 4 | 18 | 1.00 (0.81, 1.00) | 0.30 (0.27, 0.33) | 0.02 (0.01, 0.04) | 1.00 (0.99, 1.00) | 1.43 | 0 |
| IMCI pneumonia | 1 | 1040 | 98.9% | 0 | 18 | 1.00 (0.81, 1.00) | 0.01 (0.01, 0.02) | 0.02 (0.01, 0.03) | 1.00 (0.74, 1.00) | 1.01 | 0 |
| IMCI pneumonia | 0 | 1052 | 100.0% | 0 | 18 | 1.00 (0.81, 1.00) | 0.00 (0.00, 0.00) | 0.02 (0.01, 0.03) |  | 1 |  |
| Danger signs | 3 | 31 | 2.9% | 6 | 6 | 0.33 (0.13, 0.59) | 0.98 (0.96, 0.98) | 0.19 (0.07, 0.37) | 0.99 (0.98, 0.99) | 13.79 (6.45, 29.46) | 0.68 (0.49, 0.95) |
| Danger signs | 2 | 223 | 21.2% | 5 | 11 | 0.61 (0.36, 0.83) | 0.79 (0.77, 0.82) | 0.05 (0.02, 0.09) | 0.99 (0.98, 1.00) | 2.98 (2.02, 4.39) | 0.49 (0.27, 0.87) |
| Danger signs | 1 | 699 | 66.4% | 5 | 16 | 0.89 (0.65, 0.99) | 0.34 (0.31, 0.37) | 0.02 (0.01, 0.04) | 0.99 (0.98, 1.00) | 1.35 (1.14, 1.59) | 0.33 (0.09, 1.21) |
| Danger signs | 0 | 1052 | 100.0% | 2 | 18 | 1.00 (0.81, 1.00) | 0.00 (0.00, 0.00) | 0.02 (0.01, 0.03) |  | 1 |  |

## Figure S1

Score distributions overall and by outcome. Note different y-axis scales.

Note: “no unconsc” indicates the PediCAP variable ‘lethargy or unconsciousness’ was considered as ‘lethargy’, rather than as ‘unconsciousness’ as in the primary analysis.

## Figure S2

Contribution of score components to total score across all children. See table S2 for additional information on score components.

Note: “no unconsc” indicates the PediCAP variable ‘lethargy or unconsciousness’ was considered as ‘lethargy’, rather than as ‘unconsciousness’ as in the primary analysis.

## Figure S3

Case fatality rates (CFR) and 95% confidence intervals for score components with CFR above the overall CFR for each outcome (red line)

## Figure S4

ROC-AUC for secondary outcome of death in hospital + 7 days

## Code S1

**R code to calculate performance metrics**

library(tidyverse)

library(pROC) # for calcualting AUC curves

library(gridExtra) # for formatting output

library(PRROC) # for calcualting AUCs

set.seed(1900)

#' reading in files -----------------------------------------

\# read in datasets

scores <- readRDS("03_clean_data/scores_outcomes.RDS")

\# setting vars ------------------------------------------------------------

score_vars <- c("PREPARE", "PREPARE: no unconsc", "RISC-KGMU", "PERCH", "PERCH: no unconsc", "RISC-Malawi", "RISC-Malawi: no unconsc", "RISC", "IMCI pneumonia", "Danger signs")

outcome_vars <- c("deathinhosp", "deathin7days", "readmission_or_death" )

\# calcuating AUC ----------------------------------------------

\# calculate metrics

calc_auc_boot <- function(data,

outcome,

score,

site_var = "Centre",

n_boot = 1000) {

\# -----------------------------

\# point estimates

\# -----------------------------

truth <- data[[outcome]]

preds <- data[[score]]

\# remove missing outcome or score values

keep <- !is.na(truth) & !is.na(preds)

data <- data[keep, ]

truth <- data[[outcome]]

preds <- data[[score]]

auc_val <- tryCatch({

as.numeric(

pROC::auc(

pROC::roc(

truth,

preds,

quiet = TRUE,

direction = "<"

)

)

)

}, error = function(e) NA_real_)

prauc_val <- tryCatch({

PRROC::pr.curve(

scores.class0 = preds[truth == 1],

scores.class1 = preds[truth == 0],

curve = FALSE

)$auc.integral

}, error = function(e) NA_real_)

\# -----------------------------

\# cluster bootstrap by site

\# -----------------------------

sites <- unique(data[[site_var]])

boot_stats <- purrr::map_dfr(

seq_len(n_boot),

function(i) {

if(i %% 100 == 0) {

cat("Bootstrap", i, "of", n_boot, "\n")

flush.console()

}

sampled_sites <- sample(

sites,

size = length(sites),

replace = TRUE

)

boot_dat <- purrr::map2_dfr(

sampled_sites,

seq_along(sampled_sites),

function(s, id) {

data %>%

dplyr::filter(.data[[site_var]] == s) %>%

dplyr::mutate(boot_cluster = id)

}

)

y <- boot_dat[[outcome]]

x <- boot_dat[[score]]

keep <- !is.na(y) & !is.na(x)

y <- y[keep]

x <- x[keep]

auc_b <- tryCatch({

if(length(unique(stats::na.omit(y))) < 2) {

NA_real_

} else {

as.numeric(

pROC::auc(

pROC::roc(

y,

x,

quiet = TRUE,

direction = "<"

)

)

)

}

}, error = function(e) NA_real_)

prauc_b <- tryCatch({

if(length(unique(y)) < 2) {

NA_real_

} else {

PRROC::pr.curve(

scores.class0 = x[y == 1],

scores.class1 = x[y == 0],

curve = FALSE

)$auc.integral

}

}, error = function(e) NA_real_)

tibble::tibble(

auc = auc_b,

prauc = prauc_b

)

}

)

\# remove failed bootstrap iterations

boot_stats <- boot_stats %>%

dplyr::filter(!is.na(auc))

tibble::tibble(

n = nrow(data),

events = sum(data[[outcome]] == 1, na.rm = TRUE),

auc = auc_val,

auc_lo = quantile(

boot_stats$auc,

0.025,

na.rm = TRUE

) |> unname(),

auc_hi = quantile(

boot_stats$auc,

0.975,

na.rm = TRUE

) |> unname(),

prauc = prauc_val,

prauc_lo = quantile(

boot_stats$prauc,

0.025,

na.rm = TRUE

) |> unname(),

prauc_hi = quantile(

boot_stats$prauc,

0.975,

na.rm = TRUE

) |> unname(),

n_boot_valid = nrow(boot_stats)

)

}

\# apply to all outcome-score pairs

results <- tidyr::expand_grid(

outcome = outcome_vars,

score = score_vars

) %>%

mutate(

metrics = purrr::map2(

outcome,

score,

~ calc_auc_boot(

scores,

.x,

.y,

site_var = "Centre",

n_boot = 500

)

)

) %>%

tidyr::unnest(metrics)

## References for supplementary information

1.	Rees CA, Haggie S, Florin TA. Narrative review of clinical prediction models for paediatric community acquired pneumonia. Paediatric respiratory reviews 2025.

2.	McCollum ED, Brown SP, Nkwopara E, Mvalo T, May S, Ginsburg AS. Development of a prognostic risk score to aid antibiotic decision-making for children aged 2-59 months with World Health Organization fast breathing pneumonia in Malawi: An Innovative Treatments in Pneumonia (ITIP) secondary analysis. Plos one 2019;14(6):e0214583.

3.	King C, McCollum ED, Mankhambo L, et al. Can we predict oral antibiotic treatment failure in children with fast-breathing pneumonia managed at the community level? A prospective cohort study in Malawi. PLoS One 2015;10(8):e0136839.

4.	Xu C, Tao X, Zhu J, et al. Clinical features and risk factors analysis for poor outcomes of severe community-acquired pneumonia in children: a nomogram prediction model. Frontiers in Pediatrics 2023;11:1194186.

5.	Anteneh ZA, Arega HE, Mihretie KM. Validation of risk prediction for outcomes of severe community-acquired pneumonia among under-five children in Amhara region, Northwest Ethiopia. Plos one 2023;18(2):e0281209.

6.	Valentania V, Somasetia DH, Hilmanto D, Setiabudi D, Nataprawira HMN. Modified PIRO (predisposition, insult, response, organ dysfunction) severity score as a predictor for mortality of children with pneumonia in Hasan Sadikin Hospital, Bandung, Indonesia. Multidisciplinary Respiratory Medicine 2021;16(1):735.

7.	Emukule GO, McMorrow M, Ulloa C, et al. Predicting mortality among hospitalized children with respiratory illness in Western Kenya, 2009–2012. PloS one 2014;9(3):e92968.

8.	Reed C, Madhi SA, Klugman KP, et al. Development of the Respiratory Index of Severity in Children (RISC) score among young children with respiratory infections in South Africa. PloS one 2012;7(1):e27793.

9.	Mamun GMS, Zou M, Sarmin M, et al. Derivation and validation of a clinical prediction model for risk-stratification of children hospitalized with severe pneumonia in Bangladesh. PLOS Global Public Health 2023;3(8):e0002216.
